# Long-term outcomes after different types of Horne and Tanzer olecranon fractures

**DOI:** 10.1007/s00402-020-03453-z

**Published:** 2020-04-23

**Authors:** Ralph Hasserius, Fredrik Hertervig, Per-Olof Josefsson, Jack Besjakov, Thord von Schewelov

**Affiliations:** 1 Departments of Orthopaedics, Skane University Hospital, 205 02 Malmo, Sweden; 2 Departments of Radiology, Skane University Hospital, Malmo, Sweden; 3 Department of Clinical Sciences Malmo (IKVM), Lund University, Malmo, Sweden

**Keywords:** Fractures, Olecranon, Horne and Tanzer, Long-term outcome

## Abstract

**Background:**

It is unclear whether the outcomes differ after different Horne and Tanzer types of olecranon fractures.

**Materials and methods:**

We evaluated 40 men and 55 women with isolated olecranon fractures, journals and radiographs from fracture event. The fractures were classified according to Horne and Tanzer. After a mean 19 years after the fracture events, we evaluated subjective, clinical and radiographic outcomes, using the uninjured arms as controls.

**Results:**

95% of patients with type I fractures reported, at follow-up, no differences between the elbows, 80% with type II fractures and 95% with type III fractures (*p* = 0.43). The three types of fractures had no differences in elbow range of motion or hand grip strength (when comparing injured and uninjured arms) and the proportions of individuals with radiographic elbow degenerative changes or joint space reduction were no different (all *p* > 0.05). Individuals with elbow degenerative changes had no inferior subjective elbow function compared to individuals with normal radiographs (*p* = 0.66), in contrast to those with joint space reduction compared to individuals with normal radiographs (*p* < 0.001).

**Interpretation:**

All types of Horne and Tanzer olecranon fractures have favourable long-term outcome. Elbow joint space reduction is associated with inferior subjective elbow function while degenerative changes are not.

## Introduction

Fractures of the olecranon account for around 10% of all upper extremity fractures [1–3]. This corresponds to an incidence of 1.15/10,000 person-years [1–3]. The short and medium term outcome of these fractures is generally reported as good [4–14], and the few existing long-term studies, one of which followed olecranon fractures for more than 10 years [15] and another for more than 15 years [2], support a durable outcome with time. No study has to our knowledge evaluated if the favorable long-term outcome accounts for all subtypes of olecranon fractures. Most short-term studies have only evaluated if the outcome differs after different type of surgeries [4–14]. On such study inferred that type of surgery are of more importance for the outcome than type of fracture [16], another study supporting this view when reporting no differences when comparing 78 consecutive patients with Mayo type IIA and IIB fractures [17]. The clinical and radiological outcome ought however to be evaluated in regard to different types and anatomic locations of isolated fractures in proximal ulnae, since fractures that involve mechanically loaded articular surfaces are in a long-term perspective to a greater extent associated with post-traumatic osteoarthritis and disability than extra-articular fractures [1, 18]. Furthermore, fractures distal to the triceps tendon, are more exposed to displacing forces, with the risk of developing articular diastasis, than fractures proximal to the insertion [3, 19–21]. A recent published review, also states that there are several classifications of olecranon fractures that correlate with the severity of the injury [6]. The same review also states that there is a need for more evidence to determine prognostic surgical markers for good clinical outcomes [6]. Our survey was done 1993–1994, a period when the Horne and Tanzer [22] was one of the most used classification system, the same year when the Mayo classification was presented, but still not being generally accepted as a classification system. Horne and Tanzer type I fractures include transverse intra-articular fractures at the proximal third of the olecranon articular surface or oblique extra-articular fractures that involve the process of the olecranon. Type II fractures include oblique or transverse fractures that involve the middle third of the greater sigmoid notch. Type III fractures involve the distal third of the greater sigmoid notch, with or without a coronoid fracture. The primary aim of this study was to determine if different Horne and Tanzer type of olecranon fractures have different clinical and radiographic long-term outcomes, and the secondary to evaluate whether posttraumatic radiographic elbow changes are associated with an inferior clinical outcome.

## Materials and methods

Our hospital is the only emergency hospital in the city, and thus treats all fracture patients in the region. The city had 264,937 inhabitants in 1970. As the hospital saves radiographs, referrals and reports, and has done so for the last century, it is possible to identify and reclassify old fractures. Furthermore, since all citizens in our country have a unique 10-digit personal identity number, it is possible to localize former patients, decades after an injury for long-term follow-up studies. In this study, we evaluated radiographs of all city patients who received care at the hospital for elbow fractures 1969–1979. Out of 2965 patients with elbow fractures, 315 patients had an isolated fracture of the olecranon. That is, we excluded Monteggia, Monteggia-like and transolecranon fractures and fracture dislocations.

**Fig. 1:**
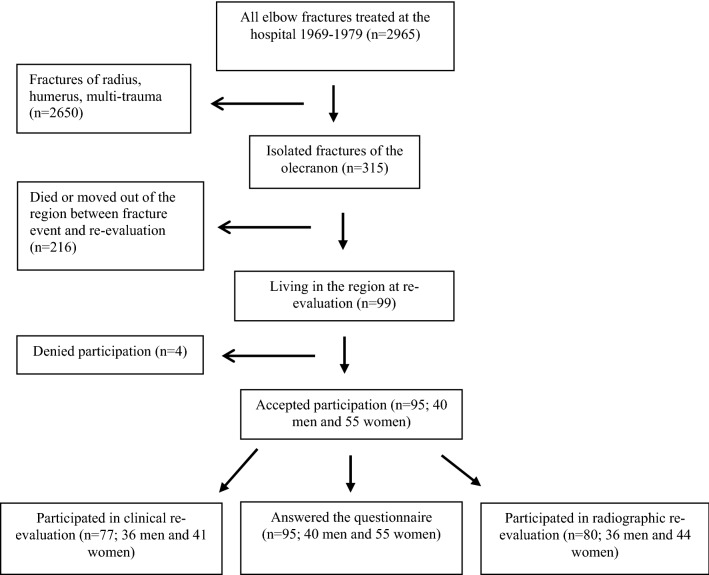
Flow-chart describing participants

**Fig. 2:**
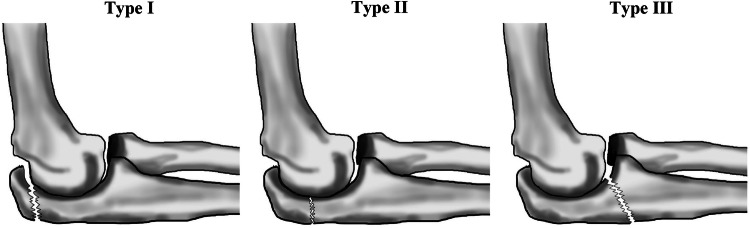
Classification of olecranon fractures according to Horne and Tanzer. Type I fractures include transverse intra-articular fractures at the proximal third of the olecranon articular surface or oblique extra-articular fractures that involves the process of the olecranon. Type II fractures include oblique or transverse fractures that involve the middle third of the greater sigmoid notch. Type III fractures involve the distal third of the greater sigmoid notch, with or without a coronoid fracture

Of the former olecranon fracture patients, 216/315 had died or relocated out of the region two decades after the fracture event. The remaining 99 patients were invited to this follow-up study. We wanted to include all consecutive patients in this study, even if it is reported that growing patients usually have a favorable outcome independent on the type of fracture and that non-operative management could be used with good outcome in most isolated displaced fractures of the olecranon in the elderly [2, 4]*.* Forty men and 55 women with a mean age of 38.6 years (range 5–77) at injury participated finally a mean 18.8 years (range 15.0–25.0) after the fracture event (Fig. 1). Primary radiographs were classified according to Horne and Tanzer [22] (Fig. 2). There were 20 type I fractures, 55 type II fractures and 20 type III fractures. Out of these fractures, 19 were displaced less than 2 mm, 57 displaced more than 2 mm and 19 comminuted (Table 1). Fifty-three patients (19 men, 34 women) had sustained their fractures due to low-energy trauma (defined as a blow to the elbow or falling from standing height or less) while 39 (20 men, 19 women) had suffered a high-energy trauma (defined as a fall from higher than 2 m or being involved in a motor vehicle accident). Information regarding trauma type was missing in three patients. The right elbows were injured in 45 and the left elbow in 50 patients.

**Table 1:** Background data in 95 patients with Horne and Tanzer type I, II or III fractures of the olecranon

	Type I (*n* = 20)	Type II (*n* = 55)	Type III (*n* = 20)
Age (years)			
At injury	35.0 ± 20.5	38.7 ± 19.5	42.0 ± 24.9
At follow-up	54.0 ± 20.5	57.5 ± 19.0	60.4 ± 23.4
Follow-up period (years)	19.0 ± 3.0	18.8 ± 2.9	18.4 ± 3.3
Gender [*n* (%)]			
Men	10 (50%)	21 (38%)	9 (45%)
Women	10 (50%)	34 (62%)	11 (55%)
Trauma type [*n* (%)]			
Low-energy	15 (75%)	26 (48%)	12 (67%)
High-energy	5 (25%)	28 (52%)	6 (33%)
Fracture displacement [*n* (%)]			
Undisplaced	6 (30%)	9 (16%)	4 (20%)
Displaced two-fragment	10 (50%)	35 (64%)	12 (60%)
Comminuted	4 (20%)	11 (20%)	4 (20%)
Primary treatment [*n* (%)]			
Instant mobilization	3 (15%)	3 (5%)	0 (0%)
Plaster	3 (15%)	8 (16%)	7 (35%)
Open reduction and internal fixation	14 (60%)	44 (79%)	13 (65%)
Current workload [*n* (%)]			
White-collar	7 (39%)	23 (46%)	7 (39%)
Blue-collar	3 (17%)	4 (8%)	1 (6%)
Retired	8 (44%)	23 (46%)	10 (55%)

Data were missing for trauma type in three patients and current work load in nine. Data are shown as mean ± SD or numbers with proportions (%)

Primary treatments included direct mobilization in six patients, immobilization with plaster cast for a mean 4.3 weeks (range 1.5–8.0 weeks) in 18 patients, open reduction and internal fixation with figure-of-eight cerclage [23] in 34, tension band wiring technique [24] in 32 and operations with other surgical techniques in five patients (Table 1). The surgeon at call decided the treatment strategy according to the clinical routine. Patients who underwent surgery were after the operation supported with a plaster cast for mean 4.4 weeks (range 0.1–7.0). After surgery, 17 patients had a remaining diastasis in the articular surface of more than 2 mm and six individuals more than 3 mm. After the fracture had healed, 41/71 (58%) of the operated patients had a second operation, in all cases including removal of the osteosynthesis.

Subjective outcomes were at follow-up evaluated in all 95 former patients through a non-validated questionnaire [23] that compared the former injured and uninjured elbows with respect to pain at rest, pain on loading, perceived instability, perceived strength and whether there were differences between the elbows.

Objective outcomes were at follow-up evaluated in the 77 patients (36 men, 41 women), with a mean age of 37.8 years (range 5–70) at injury, who attended the clinical examination a mean 18.8 years (range 15.0–25.0) after the fracture event. Objective outcome was evaluated through clinical exams by two consultants in orthopaedics, unaware of treatments given and unaware of previous and current subjective and radiographic outcome. The clinical evaluations included examinations of both arms, with the uninjured arms serving as controls. Arm circumference was measured 10 cm above and below the tip of the olecranon. Degree of flexion and extension of elbows and wrists, pronation and supination of the forearms and the valgus/varus angle of the extended elbows were measured with a goniometer. Grip strength was measured by a Martin vigorimeter (Heinrich C. Ulrich, Werkstätten für Medizinmechanik^®^, Ulm-Donau, Germany).

Radiographic outcomes were at follow-up evaluated in the 80 patients (36 men, 41 women), with a mean age of 37.8 years (range 5–70) at injury, who attended the radiographic exam a mean 18.9 years (range 15.0–25.0) after the fracture event. Radiographic outcome was evaluated through anteroposterior and lateral projections of both elbows and forearms, with the uninjured arms serving as controls. All radiographs were reviewed by one radiologist, uninvolved in the treatment of the patients, unaware of the type of index fracture and unaware of the clinical outcome. Degenerative changes were defined as any signs of subchondral sclerosis, subchondral cysts, osteophytes or joint space narrowing greater than 1 mm compared to the uninjured elbow. We defined joint space narrowing above 1 mm as a radiographic sign of osteoarthritis [23].

Descriptive data are presented as numbers, proportions (%), mean ± standard deviations (SD) and mean with range, and the inferential uncertainty as mean with 95% confidence intervals (95% CI). Group comparisons were conducted with Fisher’s exact test, Chi squares test, paired Student’s *t* test and unpaired Student’s *t* test. A *p* < 0.05 is regarded as a statistically significant difference. The study was approved by the Ethical Committee in our region (Diary number: LU-345-95) and performed in accordance with the recommendations in the Declaration of Helsinki.

## Results

Of former patients, 81/95 (85%) experienced no subjective difference in former fractured and uninjured elbows, 11 (12%) reported slightly inferior function and 3 (3%) markedly inferior function. There were no statistically significant differences in function (*p* = 0.43) or objective differences in injured to uninjured arm deficits (all *p* > 0.05) when the three types of Horne and Tanzer fractures were compared (Tables 2, 3).

**Table 2:** Subjective outcome in 95 patients a mean 19 years after a Horne and Tanzer type I, II or III fracture of the olecranon

	Type I (*n* = 20)	Type II (*n* = 55)	Type III (*n* = 20)	*p* value
Elbow pain at rest				
Yes	0 (0%)	3 (5%)	1 (5%)	0.57
No	20 (100%)	52 (95%)	19 (95%)	
Elbow pain at load				
Yes	1 (5%)	8 (15%)	1 (5%)	0.28
No	18 (95%)	47 (85%)	19 (95%)	
Elbow instability				
Yes	0 (0%)	1 (2%)	0 (0%)	0.70
No	20 (100%)	54 (98%)	19 (100%)	
Elbow weakness				
Yes	1 (5%)	7 (13%)	0 (0%)	0.17
No	19 (95%)	48 (87%)	20 (100%)	
Status compared to uninjured elbow				
No difference	18 (90%)	44 (80%)	19 (95%)	0.43
Slightly inferior	2 (10%)	8 (15%)	1 (5%)	
Markedly inferior	0 (0%)	3 (5%)	0 (0%)	

Data were missing for pain at load in one and instability in one patient. Data are shown as numbers and proportions (%)

**Table 3:** Objective outcome in 77 patients a mean 19 years after a Horne and Tanzer type I, II or III fracture of the olecranon, with comparison between formerly uninjured and formerly fractured upper extremities

	Type I (*n* = 16)			Type II (*n* = 46)			Type III (*n* = 15)		
	Fractured arm	Uninjured arm	Uninjured to fractured arm difference	Fractured arm	Uninjured arm	Uninjured to fractured arm difference	Fractured arm	Uninjured arm	Uninjured to fractured arm difference
Elbow flexion (°)	141 ± 4	141 ± 5	0.0 (− 1.4, 1.4)	140 ± 11	143 ± 6	**2.7 (0.1, 5.3)**	140 ± 9	141 ± 8	0.7 (−1.6, 3.0)
Elbow extension (°)	−4 ± 7	−4 ± 9	0.0 (− 4.2, 4.2)	−5 ± 11	0 ± 6	**4.9 (2.2, 7.6)**	−2 ± 9	−1 ± 7	1.3 (−3.2, 5.8)
Forearm pronation (°)	84 ± 9	85 ± 8	0.6 (− 0.7, 2.0)	83 ± 12	84 ± 9	1.4 (−1.1, 3.9)	87 ± 6	86 ± 6	−1.0 (−3.1, 1.1)
Forearm supination (°)	83 ± 19	83 ± 16	0.0 (− 1.9, 1.9)	83 ± 15	86 ± 6	2.9 (−1.1, 7.0)	84 ± 12	87 ± 6	2.7 (−4.3, 9.7)
Elbow valgus angle (°)	8 ± 4	9 ± 7	1.2 (− 1.4, 3.9)	9 ± 7	7 ± 5	**−1.8 (−3.2, −0.4)**	8 ± 6	8 ± 6	−0.3 (−1.0, 0.4)
Wrist flexion (°)	66 ± 13	68 ± 10	2.0 (−3.5, 7.5)	67 ± 15	68 ± 12	1.4 (−0.7, 3.5)	69 ± 10	68 ± 11	−1.6 (−3.7, 0.3)
Wrist extension (°)	68 ± 10	67 ± 11	− 1.3 (− 3.5, 0.9)	62 ± 14	62 ± 16	0.3 (−2.1, 2.8)	60 ± 11	60 ± 11	−0.1 (−1.6, 1.4)
Circumference upper arm (cm)	27.1 ± 2.7	27.1 ± 2.8	0.0 (− 0.3, 0.3)	27.2 ± 3.0	27.3 ± 2.9	0.1 (−0.0, 0.3)	26.8 ± 3.3	26.6 ± 3.2	−0.2 (−0.4, 0.1)
Circumference forearm (cm)	24.8 ± 2.7	24.7 ± 2.7	− 0.1 (− 0.3, 0.3)	24.9 ± 3.0	24.9 ± 3.0	0.1 (−0.2, 0.3)	24.5 ± 3.3	24.3 ± 3.0	−0.2 (−0.5, 0.2)
Grip strength (kp/cm^2^)	0.76 ± 0.33	0.79 ± 0.35	0.03 (− 0.03, 0.10)	0.73 ± 0.41	0.76 ± 0.39	0.02 (−0.01, 0.06)	0.83 ± 0.32	0.80 ± 0.33	−0.02 (−0.09, 0.04)

Data are provided as mean ± SD and in comparisons of the difference between the arms as mean (95% CI). Statistically significant differences between injured and uninjured extremities are highlighted in bold text. No statistically significant differences were found in arm differences between the three types of fractures

The three individuals with markedly inferior function had all sustained type II fractures. Two of these patients had been operated with figure-of-eight cerclage and one with tension band wiring technique. One of these patients had a postoperative articular diastasis exceeding 3 mm while the two others had no articular diastasis, all had at follow-up 20° or more deficits in elbow ROM (20, 40 and 80°, respectively), one a joint space reduction exceeding 1 mm while the two others had no joint space reduction.

All three types of Horne and Tanzer fractures were associated with degenerative changes but not with joint space reduction (comparing injured vs. uninjured elbows) (all *p* > 0.05) (Table 4). There were no differences between the three types of fractures in either the proportion of individuals with degenerative changes or the proportion of individuals with joint space reduction (all *p* > 0.05) (Table 4). There was no difference in subjective elbow function between elbows with and without degenerative changes (*p* > 0.05), while the subjective function was inferior in those with joint space reduction compared to those with no joint space reduction (*p* < 0.001) (Table 5).

**Table 4:** Radiographic outcome in 80 examined patients a mean 19 years after a Horne and Tanzer type I, II or III fracture of the olecranon

	Type I (*n* = 17)			Type II (*n* = 49)			Type III (*n* = 14)			Comparison fractured elbows
	Fractured	Uninjured	*p* value	Fractured	Uninjured	*p* value	Fractured	Uninjured	*p* value	*p *value
Osteophytes										
Yes	4 (24%)	2 (12%)	0.32	17 (35%)	3 (6%)	** < 0.001**	6 (43%)	0 (0%)	**0.008**	0.51
No	13 (76%)	15 (88%)		32 (65%)	46 (94%)		8 (57%)	14 (100%)		
Cysts										
Yes	2 (12%)	0 (0%)	0.24	20 (41%)	1 (2%)	** < 0.001**	5 (36%)	0 (0%)	**0.02**	0.09
No	15 (88%)	17 (100%)		29 (59%)	48 (98%)		9 (64%)	14 (100%)		
Subchondral sclerosis										
Yes	12 (71%)	6 (35%)	**0.04**	38 (78%)	3 (6%)	** < 0.001**	11 (79%)	0 (0%)	** < 0.001**	0.82
No	5 (29%)	11 (65%)		11 (22%)	46 (94%)		3 (21%)	14 (100%)		
Reduced joint space										
Yes	1 (6%)	0 (0%)	0.50	3 (6%)	1 (2%)	0.29	1 (7%)	0 (0%)	0.50	0.99
No	16 (94%)	17 (100%)		44 (94%)	46 (98%)		13 (93%)	14 (100%)		
Any degenerative changes										
Yes	12 (71%)	6 (35%)	**0.04**	40 (82%)	3 (6%)	** < 0.001**	12 (86%)	0 (0%)	** < 0.001**	0.52
No	5 (29%)	11 (100%)		9 (18%)	46 (94%)		2 (14%)	14 (100%)		

Data was missing for joint space height in two patients. Statistically significant differences are highlighted in bold text

**Table 5:** Relation between degenerative radiographic changes and joint space reduction and clinical symptoms in elbows a mean 19 years after a Horne and Tanzer type I, II or III fracture of the olecranon

	Joint degeneration			Joint space reduction		
	Degenerative elbow changes (*n* = 64)	No degenerative elbow changes (*n* = 16)	*p*-value	Joint space reduction (*n* = 5)	No joint space reduction (*n* = 75)	*p* value
Elbow pain at rest						
Yes	4 (6%)	0 (0%)	0.30	1 (20%)	3 (4%)	0.12
No	60 (94%)	16 (100%)		4 (80%)	70 (96%)	
Elbow pain at load						
Yes	8 (13%)	2 (13%)	0.98	2 (40%)	8 (11%)	0.06
No	55 (87%)	14 (87%)		3 (60%)	64 (9%)	
Elbow instability						
Yes	1 (2%)	0 (0%)	0.61	0 (0%)	1 (1%)	0.79
No	62 (98%)	16 (100%)		5 (100%)	71 (99%)	
Elbow weakness						
Yes	8 (12%)	1 (6%)	0.48	2 (40%)	7 (10%)	**0.03**
No	56 (88%)	15 (93%)		3 (60%)	66 (90%)	
Status compared to uninjured elbow						
No difference	52 (81%)	14 (87%)	0.66	1 (20%)	63 (86%)	** < 0.001**
Slightly inferior	9 (14%)	2 (13%)		3 (60%)	8 (11%)	
Markedly inferior	3 (5%)	0 (0%)		1 (20%)	2 (3%)	

Data are shown as numbers and proportions (%). Statistically significant differences highlighted in bold text

## Discussion

The long-term outcome of isolated fractures of the olecranon seems to be favourable, with no subjective, objective or radiographic differences between the three types of Horne and Tanzer fractures. Only 3% of the former fracture patients rated their formerly fractured elbow markedly inferior compared to the uninjured arm, and only 4% had ROM deficits 20° or more. These long-term data support previous short- and medium-term reports of favourable outcome 1–6 years after the fracture [25–28]. Our study also shows that the influence of ROM after a fracture of the olecranon is usually minor, and this supports the view of Horne and Tanzer [22] that the subjective impact of reduced ROM is of no concern as long as the patient can reach the face. Our study also found that radiographic degenerative changes after olecranon fractures are common but of no clinical relevance, while elbow joint space reduction is rarer but with clinical implications.

Horne and Tanzer [22] reported in 100 surgically treated patients with an olecranon fracture that most patients had good clinical outcome a mean 2.5 years after surgery. The best result was found in those with a fracture in the proximal or middle third of the trochlear notch, while surgical method did not affect the outcome. The view was opposed by Rommens et al. [1], following 48 patients with olecranon fractures, concluding that the different types of Horne and Tanzer fractures had no predictive value in the short term perspective for objective outcomes. One weakness of this study was that no subjective outcomes were presented [1]. Our data, now also with subjective and radiographic outcomes included, support the view of Rommens et al. [1] and add knowledge by showing that the conclusions remain in a long-term perspective and also that there are no differences in subjective, objective or radiographic outcomes when comparing the three types of Horne and Tanzer fractures.

Since most olecranon fractures affect the articular surface, and since intra-articular fractures are associated with post-traumatic osteoarthritis and disability [1, 18], there is a need to follow patients with olecranon fractures for a long period. We found in our study that a majority of former patients had radiographic degenerative changes in the formerly fractured elbows. These data support reports by Gartsman et al. [29] who found that 20% of patients had elbow degenerative changes a mean 3.6 years after surgery of an olecranon fracture. Our study adds knowledge by showing that radiographic degenerative changes occur with similar proportions after all types of Horne and Tanzer fractures, and that the changes are of no clinical relevance within all three types of fractures. We also found that the proportion of individuals with elbow joint space reduction did not differ between the three types of fractures, but that joint space reduction is associated with inferior subjective outcomes.

Several reports infer that postoperative diastasis or malreduction is associated with inferior outcomes. Murphy et al. [30] reported in 38 patients with an isolated olecranon fractures a mean 3 years after an operation that there was an inferior outcome in patient with a fracture involving > 60% of the articular surface and a postoperative diastasis of 2 mm or greater. Eriksson et al. [28] also inferred that a postoperative diastasis of 2 mm or greater was associated with symptomatic osteoarthritis and disability. Our data oppose these inferences since we found 17 individuals with a postoperative diastasis exceeding 2 mm but only 3/95 with markedly inferior elbow function. Furthermore, only 1/3 patients with markedly inferior elbow function had a postoperative diastasis exceeding 2 mm. The differences compared to the cited studies [28, 30] may be attributable to the inclusion of other elbow injuries and other treatment strategies than in our cohort.

The strengths of our study include the large sample size, the longest follow-up period hitherto reported, and that as many as 95/99 of those invited attended. The fact that all citizens with an olecranon fracture during a defined period were included makes this a population-based study. The inclusion of subjective, objective and radiographic follow-up data is another strength of the study, as is evaluation by researchers uninvolved in the treatment or unaware of the outcome when conducting the evaluations. Other strengths include fracture classification through original radiographs, and the fact that outcome is reported, not as a mixture, but for each specific type of Horne and Tanzer fracture. Weaknesses include the non-validated questionnaire and the lack of structured clinical data from the fracture event. It would have been an advantage to have elbow strength measured by a strain gauge torque sensor. Another weakness is that the lateral and/or anteroposterior X-rays were executed following the clinical routine, making identification of the true joint space height difficult to address. It had also been an advantage to have fractures classified according to the Mayo classification, and including more than 20 patients with type I and III fractures, thereby reducing the risk of making type II errors.

In conclusion, there is in general a favourable long-term outcome of all types of isolated Horne and Tanzer olecranon fractures, with no different long-term outcome between the three sub-types. Postoperative elbow joint space reduction, but not radiographic degenerative changes, is associated with inferior subjective outcome.
